# *MTHFR* C677T and A1298C polymorphism’s effect on risk of colorectal cancer in Lynch syndrome

**DOI:** 10.1038/s41598-023-44120-8

**Published:** 2023-11-01

**Authors:** Mariann Unhjem Wiik, Mia Negline, Vidar Beisvåg, Matthew Clapham, Elizabeth Holliday, Nuria Dueñas, Joan Brunet, Marta Pineda, Nuria Bonifaci, Stefan Aretz, Hannah Klinkhammer, Isabel Spier, Claudia Perne, Andreas Mayr, Laura Valle, Jan Lubinski, Wenche Sjursen, Rodney J. Scott, Bente A. Talseth-Palmer

**Affiliations:** 1 Research Unit, Ålesund Hospital, Møre and Romsdal Hospital Trust, Ålesund, Norway; 2 Department of Medicine, Ålesund Hospital, Møre and Romsdal Hospital Trust, Ålesund, Norway; 3 Department of Biological Sciences, Faculty of Natural Sciences, Norwegian University of Science and Technology (NTNU), Trondheim, Norway; 4 School of Biomedical Science and Pharmacy, College of Health, Medicine and Wellbeing, University of Newcastle and Hunter Medical Research Institute, Newcastle, Australia; 5 Department of Clinical and Molecular Medicine, Norwegian University of Science and Technology, 7491 Trondheim, Norway; 6 St. Olav’s University Hospital, Central Staff, 7006 Trondheim, Norway; 7 School of Medicine and Public Health, College of Health, Medicine and Wellbeing, University of Newcastle and Hunter Medical Research Institute, Newcastle, Australia; 8 Hereditary Cancer Program, Catalan Institute of Oncology, Hospitalet de Llobregat, Barcelona, Spain; 9 Oncobell Program, IDIBELL, Hospitalet de Llobregat, Barcelona, Spain; 10 Centro de Investigación Biomédica en Red de Cáncer (CIBERONC), Madrid, Spain; 11 Institute of Human Genetics, Medical Faculty, University of Bonn, Bonn, Germany; 12 Center for Hereditary Tumor Syndromes, University Hospital Bonn, Bonn, Germany; 13 Institute for Medical Biometry, Informatics and Epidemiology, University Hospital Bonn, Bonn, Germany; 14 Institute for Genomic Statistics and Bioinformatics, University Hospital Bonn, Bonn, Germany; 15 International Hereditary Cancer Center, Department of Genetics and Pathology, Pomeranian Medical University, Szczecin, Poland; 16 Department of Medical Genetics, St Olavs University Hospital, Trondheim, Norway; 17 Department of Molecular Genetics, NSW Health Pathology, John Hunter Hospital, Newcastle, NSW Australia; 18 NSW Health Pathology, Newcastle, NSW Australia

**Keywords:** Gastrointestinal cancer, Genetics

## Abstract

Lynch syndrome (LS) is characterised by an increased risk of developing colorectal cancer (CRC) and other extracolonic epithelial cancers. It is caused by pathogenic germline variants in DNA mismatch repair (MMR) genes or the *EPCAM* gene, leading to a less functional DNA MMR system. Individuals diagnosed with LS (LS individuals) have a 10–80% lifetime risk of developing cancer. However, there is considerable variability in the age of cancer onset, which cannot be attributed to the specific MMR gene or variant alone. It is speculated that multiple genetic and environmental factors contribute to this variability, including two single nucleotide polymorphisms (SNPs) in the *methylenetetrahydrofolate reductase* (*MTHFR*) gene: C677T (rs1801133) and A1298C (rs1801131). By decreasing MTHFR activity, these SNPs theoretically reduce the silencing of DNA repair genes and increase the availability of nucleotides for DNA synthesis and repair, thereby protecting against early-onset cancer in LS. We investigated the effect of these SNPs on LS disease expression in 2,723 LS individuals from Australia, Poland, Germany, Norway and Spain. The association between age at cancer onset and SNP genotype (risk of cancer) was estimated using Cox regression adjusted for gender, country and affected MMR gene. For A1298C (rs1801131), both the AC and CC genotypes were significantly associated with a reduced risk of developing CRC compared to the AA genotype, but no association was seen for C677T (rs1801133). However, an aggregated effect of protective alleles was seen when combining the alleles from the two SNPs, especially for LS individuals carrying 1 and 2 alleles. For individuals with germline pathogenic variants in *MLH1,* the CC genotype of A1298C was estimated to reduce the risk of CRC significantly by 39% (HR = 0.61, 95% CI 0.42, 0.89, *p* = 0.011), while for individuals with pathogenic germline *MSH2* variants, the AC genotype (compared to AA) was estimated to reduce the risk of CRC by 26% (HR = 0.66, 95% CI 0.53, 0.83, *p* = 0.01). In comparison, no association was observed for C677T (rs1801133). In conclusion, our study suggests that combining the MMR gene information with the *MTHFR* genotype, including the aggregated effect of protective alleles, could be useful in developing an algorithm that estimates the risk of CRC in LS individuals.

## Introduction

Lynch syndrome (LS) is the most common inherited condition predisposing to colorectal cancer (CRC), and individuals with this condition (LS individuals) also have an increased risk of developing other types of epithelial cancers, most commonly in the colorectum and endometrium^1–2^. A molecular genetic diagnosis of LS is established by identifying either a germline pathogenic variant in one of the DNA mismatch repair (MMR) genes *MLH1*, *MSH2*, *MSH6* or *PMS2* or an *EPCAM* deletion affecting the expression of *MSH2*^3^. Differences in lifetime risk of CRC are known, showing that carriers of pathogenic variants in *MSH6* and *PMS2* have a lower risk of developing cancer, especially CRC and at later ages of onset than those with variants in *MLH1* and *MSH2*^4–12^. Gender differences are also observed, showing that women have a lower lifetime risk of developing CRC than men^8,9, 13, 14^.

MMR proteins are responsible for the elimination of base-substitution and insertion/deletion mismatches. Impaired or lost function of one or more MMR proteins confers genetic hypermutability and a higher risk of developing several epithelial cancers throughout life^1,15^. Differences in disease expression are observed within and among families harbouring the same MMR germline variants and are believed to result from environmental and genetic risk modifiers^15–18^.

Genetic variants in the *methylenetetrahydrofolate reductase* (*MTHFR*) gene have been proposed as genetic modifiers in LS, affecting disease expression^15, 19–21^. MTHFR is a key enzyme in the folate metabolism pathway. It catalyses the reduction of 5,10-methylenetetrahydrofolate (5,10-MTHF) to 5-methyltetrahydrofolate (5-MTHF), a methyl donor that promotes DNA methylation at the expense of thymidine synthesis^20,22^**.** A shift away from thymidine synthesis may cause uracil to be misincorporated into DNA, with excision repair leading to single-strand and double-strand breaks during replication^15,19^. In individuals with defective DNA MMR, the undesirable effects of high MTHFR activity may be deleterious^15^.

There are two common single nucleotide polymorphisms (SNPs) in the *MTHFR* gene, C677T (rs1081133) and A1298C (rs1081131), both known to reduce MTHFR activity, that have been suggested to protect against the development of cancer in LS individuals^20,23, 24^. The lower MTHFR enzyme activity is hypothesised to reduce the misincorporation of uracil into DNA, reducing the double-strand breaks needing to be repaired, thus causing the protective effect shown in cancer development.

Through international collaboration, we were able to analyse *MTHFR* C677T and A1298C in 2,723 LS individuals and investigate their association with age at cancer onset and the risk of developing CRC and any LS-related cancer.

## Materials and methods

Our sample cohort consists of Australian, Polish, German, Norwegian and Spanish LS individual samples recruited from diagnostic laboratories or family cancer clinics, all carrying pathogenic or likely pathogenic germline MMR variants. The study complies with the ethical considerations and approvals for each separate sample cohort in the respective country: the Hunter New England Research Ethics Committee (Australia), the ethics committee of the Pomeranian Academy of Medicine (Poland), the ethics committee of the University Hospital Bonn, the Regional Committees for Medical and Health Research Ethics (Norway) and the IDIBELL Ethics Committee (Spain)—all experiments were performed in accordance with institutional guidelines and regulations. Written informed consent was obtained from all participants, which for participants under the age of 18 years was their parent or guardian.

### Sample cohort

A total of 2,723 LS individual samples with appropriate clinical information available were included in the current international study from five different countries: 680 LS individuals from Australia, 410 from Poland, 557 from Germany, 204 from Norway and 872 from Spain. Demographic data is shown in Tables 1A and 1B. The sample cohort was split in two for analysis purposes depending on whether the LS individual with a cancer diagnosis was diagnosed with CRC or any other LS-related cancer (LS cancer). LS cancer in this context refers to CRC and any extra-colonic epithelial cancer associated with LS, including cancers of the uterine, stomach, liver, kidney, ovaries, brain, pancreas, and certain types of skin cancers.

**Table 1:** Displays demographic data from combined sample cohorts. (A) Displays demographics for the studied LS cohort (rs1801131 and rs1801133), while (B) Displays demographics for the five countries separately.

1A	Demographics of the combined sample								
	Samples	Mean age*	Gender**		Mutated MMR gene				
	n	Years (range)	Male (%)	Female (%)	*MLH1* (%)	*MSH2* (%)	*MSH6* (%)	*PMS2 (%)*	*EPCAM (%)*
LS cancer***	1427	45 (11–83)	652 (46)	775 (54)	573 (40)	559 (39)	226 (16)	45 (3)	24 (2)
CRC	1103	44 (13–87)	581 (53)	522 (47)	483 (44)	425 (39)	135 (12)	37 (3)	23 (2)
Cancer-free	1169	45 (12–93)	502 (43)	666 (57)	424 (36)	360 (31)	291 (25)	80 (7)	13 (1)
Total	2596								

1B^¶^	Demographics of the sample presented by country								
	Samples	Mean age**	Gender **		Mutated MMR gene				
	n	Years (range)	Male (%)	Female (%)	*MLH1* (%)	*MSH2* (%)	*MSH6* (%)	*PMS2 (%)*	*EPCAM (%)*
LS cancer AU	286	45 (19–81)	122 (43)	164 (57)	98 (34)	129 (45)	56 (20)	3 (1)	0 (0)
LS cancer PL	199	45 (18–71)	74 (37)	125 (63)	102 (51)	73 (37)	14 (7)	0 (0)	10 (5)
LS cancer NO	70	52 (26–77)	24 (34)	46 (66)	11 (16)	21 (30)	19 (27)	18 (26)	1 (1)
LS cancer GE	399	40 (11–71)	229 (57)	170 (43)	162 (41)	193 (48)	33 (8)	11 (3)	0 (0)
LS cancer ES	474	47 (21–79)	203 (43)	271 (57)	200 (42)	143 (30)	104 (22)	13 (3)	13 (3)
Total	1427								
CRC AU	206	44 (19–75)	101 (49)	105 (51)	84 (41)	93 (45)	27 (13)	2 (1)	0 (0)
CRC PL	159	43 (18–69)	70 (44)	89 (56)	85 (54)	54 (34)	10 (6)	0 (0)	10 (6)
CRC NO	41	54 (26–87)	18 (44)	23 (56)	7 (17)	11 (27)	7 (17)	15 (37)	1 (2)
CRC GE	357	41 (13–75)	214 (60)	143 (40)	148 (41)	172 (48)	27 (8)	10 (3)	0 (0)
CRC ES	340	47 (21–83)	178 (52)	162 (48)	159 (47)	95 (28)	64 (19)	10 (3)	12 (3)
Total	1103								
Cancer-free AU	297	40 (16–81)	117 (40)	178 (60)	111 (37)	109 (37)	76 (26)	0 (0)	0 (0)
Cancer-free PL	195	39 (13–73)	76 (39)	119 (61)	113 (58)	65 (33)	12 (6)	0 (0)	5 (3)
Cancer-free NO	122	46 (16–89)	52 (43)	70 (57)	11 (9)	22 (18)	48 (39)	40 (33)	1 (1)
Cancer-free GE	156	40 (12–80)	72 (46)	84 (54)	51 (33)	83 (53)	16 (10)	6 (4)	0 (0)
Cancer-free ES	399	45 (18–93)	184 (46)	215 (54)	138 (35)	81 (20)	139 (35)	34 (8)	7 (2)
Total	1169								

*Average age for LS individuals with CRC/LS and average age at last follow-up for cancer-free LS individuals. **One LS individual had no gender identified and was excluded when analysing gender (cancer-free AU group). ¶ AU = Australia, PL = Poland, NO = Norway, GE = Germany, ES = Spain.

### Genotyping

#### Australian and Polish samples

DNA samples were amplified under universal conditions using the Applied Biosystem® 7500 Real-Time (RT) PCR System (Applied Biosystems, Foster City, Ca, USA). Post-PCR allelic discrimination was performed using TaqMan® SNP Genotyping Assays (ThermoFisher Scientific) for *C677T* (rs1801133, assay ID: C___1202883_20) and *A1298C* (rs1801131, assay ID: C____850486_20). Each reaction mixture contained 0.125 µL 40 × Assay Mix, 2.5µL TaqMan® Universal PCR master mix, 1 µL DNA and Milli-Q® water to make up a final volume of 5 µL. Thermal cycling conditions were set at 60 °C for 1 min, 95℃ for 10 min, 60 cycles of 95 °C for 15 s and 60 °C for 1 min. Positive controls for each SNP genotype were used to ensure the quality of PCR performance, while no template controls (NTCs) monitored for the contamination of reagents.

#### German samples

Leukocyte-derived DNA was genotyped with the Illumina Infinium Global Screening Array (GSA) v3.0 (Illumina, Inc., San Diego, CA, USA) designed by the Global Screening Array Consortium using a semiautomated protocol. All laboratory procedures were performed in accordance with the manufacturer's instructions. Illumina raw intensity files were uploaded with the Illumina GSA manifest and cluster file into the GenomeStudio software, and genotypes were subsequently exported to PLINK format.

#### Norwegian samples

SNP genotyping for the two variants was performed using TaqMan® assays (ThermoFisher Scientific) and TaqPath ProAmp Master Mix (Applied Biosystems, ThermoFisher Scientific) according to the manufacturer’s instructions, with minor modifications. The two TaqMan Assays included SNP ID: rs1081131 (A/C Chr.3: 3739758 on GRCh38) and rs1801133 (G/A Chr.1: 11796321 on GRCh38) (ThermoFisher Scientific catalogue nr 4351376). In brief, approximately 0.75 ng DNA was used as input for the 10 µl SNP TaqMan assays run in 384-well plates. A master mix was prepared, containing 5.0 µl TaqPath ProAmp Master Mix, 0.25 µl TaqMan SNP Genotyping Assay (40X), 3 µl genomic DNA or NTC and 1.75 µl water.

The SNP genotyping assay was performed on a real-time PCR instrument (QuantStudio™ 5 Real-Time PCR System, Applied Biosystems, Thermo Fisher Scientific) under the following conditions: Pre-read (60 °CC for 30 s), initial denature/enzyme activation (95 °C for 5 min), cycling for 40 cycles (95 °C for 15 s, 60 °C for 30 s and 60 °C for 60 s) and post-read (60 °C for 30 s). SNP genotypes were obtained by the QuantStudio™ 5 Real-Time PCR System software.

#### Spanish samples

Leukocyte-derived DNA samples were genotyped with the Illumina Global Screening Array-24 v2.0 and v3.0 designed by the Global Screening Array Consortium (GSA). Samples were genotyped at once (24 samples/array). As internal controls, 23 unique samples belonging to the HapMap project were also included in duplicate to measure the experiment's reproducibility. Genotyping was performed at CEGEN (Centro Nacional de Genotipado, Instituto de Salud Carlos III, Spain).

### Statistics

Statistical analyses were performed using R version 4.1.1 (2021-08-10) (R Foundation for Statistical Computing, Vienna, Austria). Pearson’s Chi-square test was used to evaluate deviation from the expected Hardy–Weinberg equilibrium using a web-based program (http://www.dr-petrek.eu/documents/HWE.xls). For each SNP, variation in age at cancer onset by genotype was examined using Kaplan–Meier plots. Cancer-free individuals were censored at their age at last follow-up. Kaplan–Meier survival curves stratified by genotype are provided with p-values from log-rank tests assessing whether age at cancer onset differed by genotype.

In the total sample, the association between SNP genotype and age at cancer onset (risk of cancer) was analysed using a Cox proportional hazards gamma shared frailty model to allow for the relatedness of some individuals within a single-family group. Two models were provided: a crude model containing genotype only and a model additionally adjusted for gender, country and gene.

The risk of cancer was also estimated for each SNP by genotype and gene (excluding individuals with pathogenic variants in *PMS2* or *EPCAM* due to low sample numbers in the rare genotypes) using the Cox proportional hazard gamma shared frailty model as above. Two models were used: a crude model containing gene and genotype and their interaction, and a model additionally including gender and country as covariates. Hazard ratios, 95% confidence intervals and p-values were provided.

In addition, Kaplan–Meier and Cox proportional hazards gamma analysis was performed to explore the relationship between the number of protective alleles for both SNPs and age at cancer onset and cancer risk (aggregated effect of protective alleles). The protective alleles were C for A1298C (rs1801131) and T for C677T (rs1801133).

*P*-values less than 0.025 were considered statistically significant after applying a Bonferroni correction for the two SNPs analysed.

## Results

The analysis included 2,723 individuals with a molecular genetic diagnosis of LS, carrying pathogenic or likely pathogenic variants in *MLH1*, *MSH2*, *MSH6*, *PMS2* or *EPCAM* (see Table 1A for LS individual demographics). Of these, 127 samples were excluded from the study due to insufficient DNA quantity for genotyping or missing/undetermined genotyping information for both SNPs. Of the samples with informative genotyping data, three had missing/failed information for A1298C and 14 for C677T, making the sample size 2,593 for A1298C (rs1801131) and 2,582 for C677T (rs1801133). Demographics of the sample by country and genotypes for the two SNPs are shown in Tables 1B and 2, respectively. Genotype distributions were consistent with Hardy–Weinberg equilibrium for A1298C (rs1801131) (*p* = 0.126) and C677T (rs1801133) (*p* = 0.099).The mean age of cancer onset in this sample population is 47 years (54 years for *MSH6* and 44 years for both *MLH1* and *MSH2* variant carriers).

**Table 2:** Genotype frequencies and percentages for the sample cohort, total LS cohort and divided by country.

*MTHFR* SNP	Combined sample cohort total n (%)	Australia total n (%)	Poland total n (%)	Norway total n (%)	Germany total n (%)	Spain total n (%)
A1298C (rs1801131)						
AA	1,251 (48)	272 (47)	186 (47)	104 (54)	235 (42)	454 (52)
AC	1,122 (43)	259 (45)	173 (44)	72 (38)	246 (44)	372 (43)
CC	220 (8.5)	50 (8.6)	35 (8.9)	16 (8.3)	73 (13)	46 (5.3)
Total	2,593 (100)	581 (100)	394 (100)	192 (100)	554 (100)	872 (100)
C677T (rs1801133)						
CC	1,037 (40)	245 (43)	178 (46)	85 (45)	234 (42)	295 (34)
CT	1,231 (48)	274 (48)	172 (44)	83 (43)	254 (46)	448 (51)
TT	314 (12)	54 (9.4)	41 (10)	23 (12)	66 (12)	130 (15)
Total	2,582 (100)	573 (100)	391 (100)	191 (100)	554 (100)	873 (100)

Overall, no significant associations (*p* < 0.025) were observed when the data set was analysed using LS cancer in LS individuals as the endpoint of analysis. Kaplan–Meier analysis showed that within all genes, LS individuals with the SNP A1298C (rs1801131) AA genotype appeared more likely to develop LS cancer earlier than individuals with genotypes AC or CC, but the difference was not statistically significant. The same was true for Cox regression analysis; LS individuals with SNP A1298C (rs1801131) genotypes AC and CC were less likely to develop LS cancer than the AA genotype. However, the difference was not significant, see Table 3. Results using CRC as the endpoint of analysis are summarised in Tables 4 and 5.

**Table 3:** Displays the results for the crude and adjusted (gender, country and gene included as covariates) regression for SNP rs1801131(A > C) and rs1801133(C > T) in the whole sample (LS-related cancer) across all genes including EPCAM and PMS2.

Crude					Adjusted	
Characteristic	CRC-free Total n (%)	LS cancer total n (%)	HR (95% CI) total n (%)	*p*-value Total n (%)	HR (95% CI) total n (%)	*p*-value total n (%)
rs1801131(A > C)						
AA	554 (44%)	697 (56%)	1.00		1.00	
AC	525 (47%)	597 (53%)	0.90 (0.79–1.02)	0.090	0.89 (0.89–1.00)	0.060
CC	88 (40%)	132 (60%)	0.98 (0.79–1.21)	0.800	0.85 (0.69–1.05)	0.140
rs1801133(C > T)						
CC	472 (46%)	565 (54%)	1.00		1.00	
CT	555 (45%)	676 (55%)	0.99 (0.87–1.13)	0.900	1.04 (0.92–1.18)	0.500
TT	134 (43%)	180 (57%)	1.01 (0.83–1.23)	> 0.900	1.04 (0.86–1.26)	0.700

Cox shared frailty regression with age to LS cancer regressed on SNP rs1801131(A > C) and rs1801133(C > T).

**Table 4:** Displays the results for the crude and adjusted (gender, country and gene included as covariates) regression for SNP rs1801131(A > C) and rs1801133(C > T) in the CRC sample across all genes including EPCAM and PMS2.

Crude					Adjusted	
Characteristic	CRC-free total n (%)	CRC total n (%)	HR (95% CI)	*p*-value	HR (95% CI)	*p*-value
rs1801131(A > C)						
AA	554 (51%)	543 (49%)	1.00		1.00	
AC	525 (53%)	459 (47%)	0.86 (0.74–0.99)	0.036	0.83 (0.72–0.96)	0.012
CC	88 (47%)	101 (53%)	0.94 (0.73–1.21)	0.6	0.78 (0.61–0.99)	0.044
rs1801133(C > T)						
CC	472 (51%)	445 (49%)	1.00		1.00	
CT	555 (52%)	517 (48%)	0.97 (0.83–1.12)	0.6	1.02 (0.88–1.18)	0.8
TT	134 (50%)	135 (50%)	1.03 (0.82–1.30)	0.8	1.11 (0.89–1.39)	0.4

Cox shared frailty regression with age to CRC cancer regressed on SNP rs1801131(A > C) and rs1801133(C > T).

**Table 5:** Displays results from Cox Regression analysis using the genotype results for A1298C (rs1801131) from the LS CRC sample cohort divided by individual MMR genes adjusted for gender and country.

Crude				Adjusted	
Gene	GT	HR (95% CI)	*p*-value	HR (95% CI)	*p*-value
*MLH1*	AA	1		1	
*MLH1*	AC	0.93 (0.76–1.14)	0.465	0.88 (0.71–1.09)	0.236
*MLH1*	CC	0.74 (0.51–1.06)	0.095	0.61 (0.42–0.89)	0.011
*MSH2*	AA	1		1	
*MSH2*	AC	0.76 (0.61–0.94)	0.013	0.74 (0.58–0.93)	0.010
*MSH2*	CC	0.99 (0.70–1.41)	0.972	0.87 (0.60–1.25)	0.446
*MSH6*	AA	1		1	
*MSH6*	AC	1.01 (0.70–1.45)	0.978	0.98 (0.67–1.44)	0.915
*MSH6*	CC	1.10 (0.57–2.15)	0.773	1.05 (0.52–2.10)	0.894

Both *PMS2* and *EPCAM* were excluded from this analysis due to low sample numbers.

### Risk of CRC

**Figure 1:**
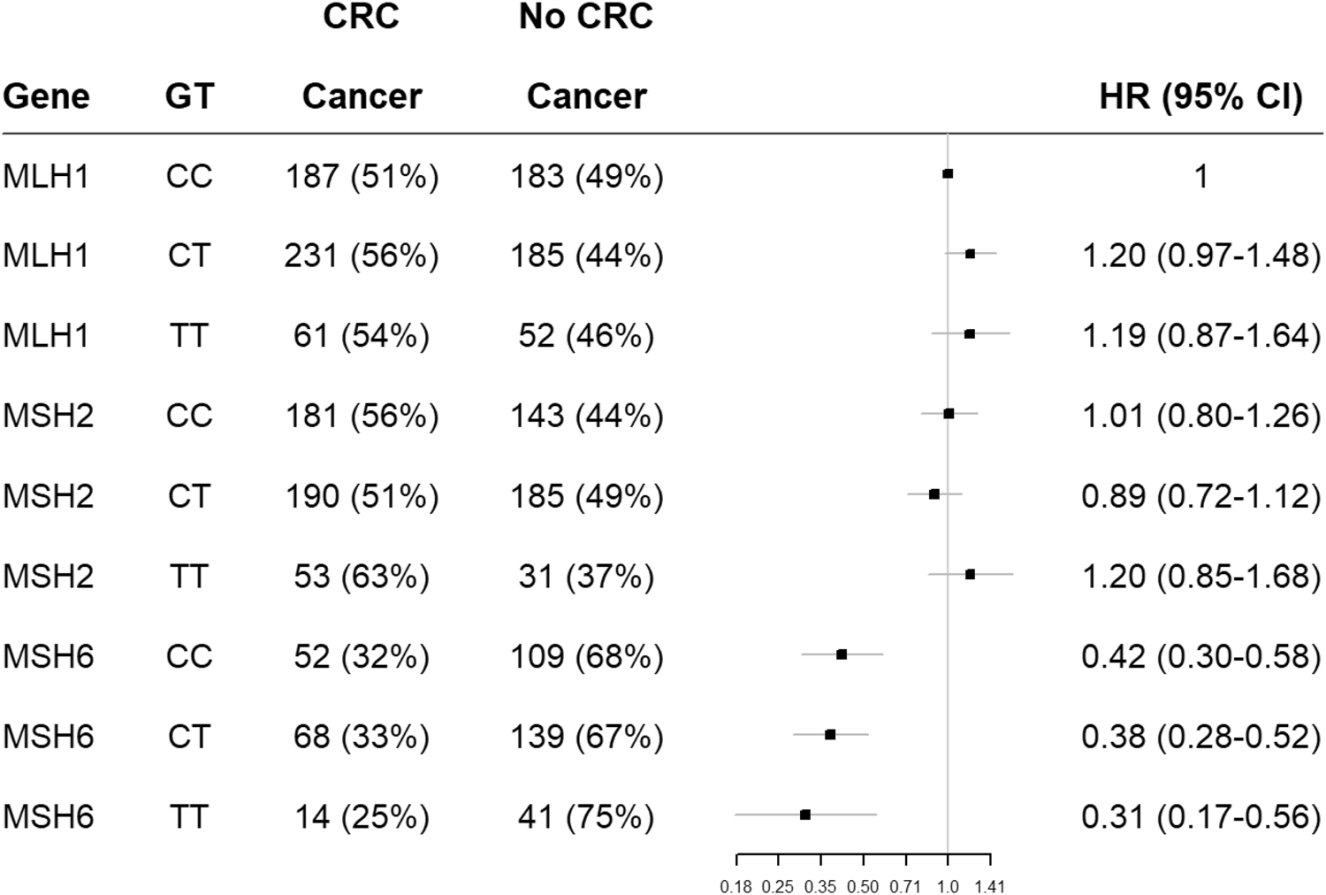
Displays C677T (rs1801133) hazard ratios for risk of CRC in MLH1, MSH2 and MSH6 pathogenic variant carriers.

**Figure 2:**
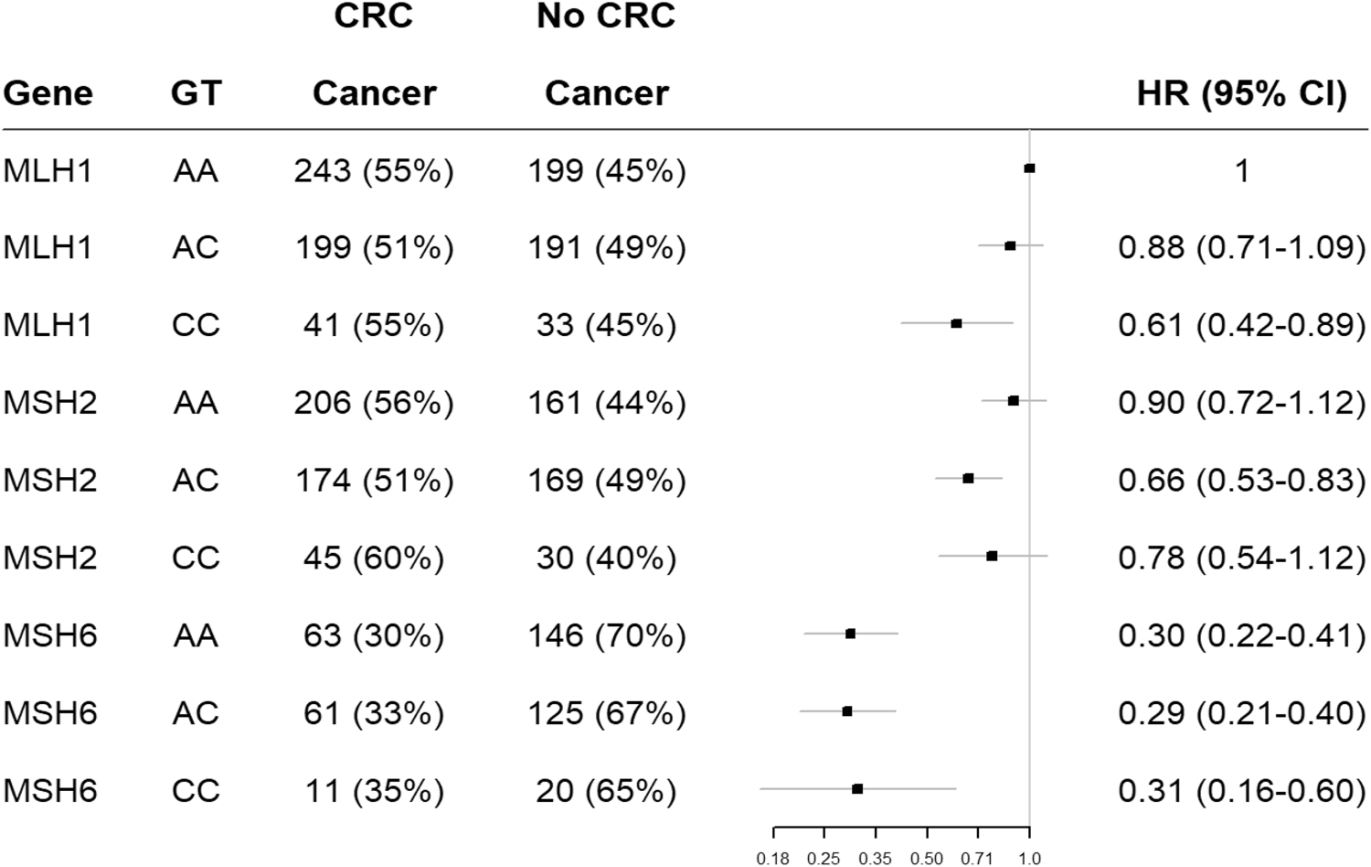
Displays A1298C (rs1801131) hazard ratios for risk of CRC in MLH1, MSH2 and MS6 pathogenic variant carriers.

As expected, individuals with germline variants in *MSH6* demonstrated a reduced risk of CRC (mean age of onset 54 years) compared to both *MLH1* and *MSH2* (both with a mean age of onset of 44 years) germline variant carriers (this is consistent with all genotypes for both SNPs in the current study), see Figs. 1 and 2. The same was observed when using LS cancer as the endpoint of analysis (data not shown).

With Cox regression analysis adjusted for gender, country of sample origin and mutated MMR gene, LS-individuals with A1298C (rs1801131) genotypes AC and CC were less likely to develop CRC than those with genotype AA (17% estimated reduction in risk; HR 0.83 (CI 0.72–0.96), *p* = 0.012 and 22% reduction in risk; HR 0.78 (CI 0.61–0.99), *p* = 0.044 respectively, see Table 4). Only the AC genotype was associated with a significant reduction in risk due to the adjusted significance threshold of 0.025. No significant difference between genotypes for C677T (rs1801133) and risk of CRC was observed, see Table 4.

In the analysis by mutated MMR gene (*PMS2* and *EPCAM* excluded due to low sample number), for individuals with germline pathogenic variants in the *MLH1* gene we observed that those with the CC genotype of A1298C (rs1801131) had a 39% lower risk of developing CRC than individuals with the AA genotype (HR 0.61 (CI 0.42–0.89), *p* = 0.011, see Table 5 and Fig. 1). No significant association was found for C677T (rs1801133) (see Table 6). Interestingly, *MSH2* variant carriers carrying the AC genotype for rs1801131 had a significantly reduced risk of CRC, with a 26% reduction compared to those with the AA genotype (HR 0.74 (CI0.58–0.93), *p* = 0.010, see Table 4 and Fig. 2) but not those with the CC genotype. Again, results were not significant for rs1801133, see Table 6.

**Table 6:** Displays results from Cox Regression analysis using the genotype results for C677T (rs1801133) from the LS-related cancer sample cohort divided by individual MMR genes adjusted for gender and country.

Crude				Adjusted	
Gene	GT	HR (95%CI)	*p*-value	HR (95%CI)	*p*-value
*MLH1*	CC	1		1	
*MLH1*	CT	0.18 (0.95–1.47)	0.135	01.20 (0.97–1.48)	0.095
*MLH1*	TT	1.15 (0.82–1.60)	0.414	1.19 (0.87–1.64)	0.277
*MSH2*	CC	1		1	
*MSH2*	CT	0.87 (0.69–1.09)	0.232	0.89 (0.71–1.11)	0.298
*MSH2*	TT	1.19 (0.84–1.70)	0.328	1.19 (0.85–1.67)	0.314
*MSH6*	CC	1		1	
*MSH6*	CT	0.92 (0.62–1.35)	0.656	0.91 (0.62–1.33)	0.624
*MSH6*	TT	0.69 (0.37–1.30)	0.251	0.74 (0.40–1.38)	0.350

Both *PMS2* and *EPCAM* were excluded from this analysis due to low sample numbers.

### Aggregated effect of combined protective alleles

**Figure 3:**
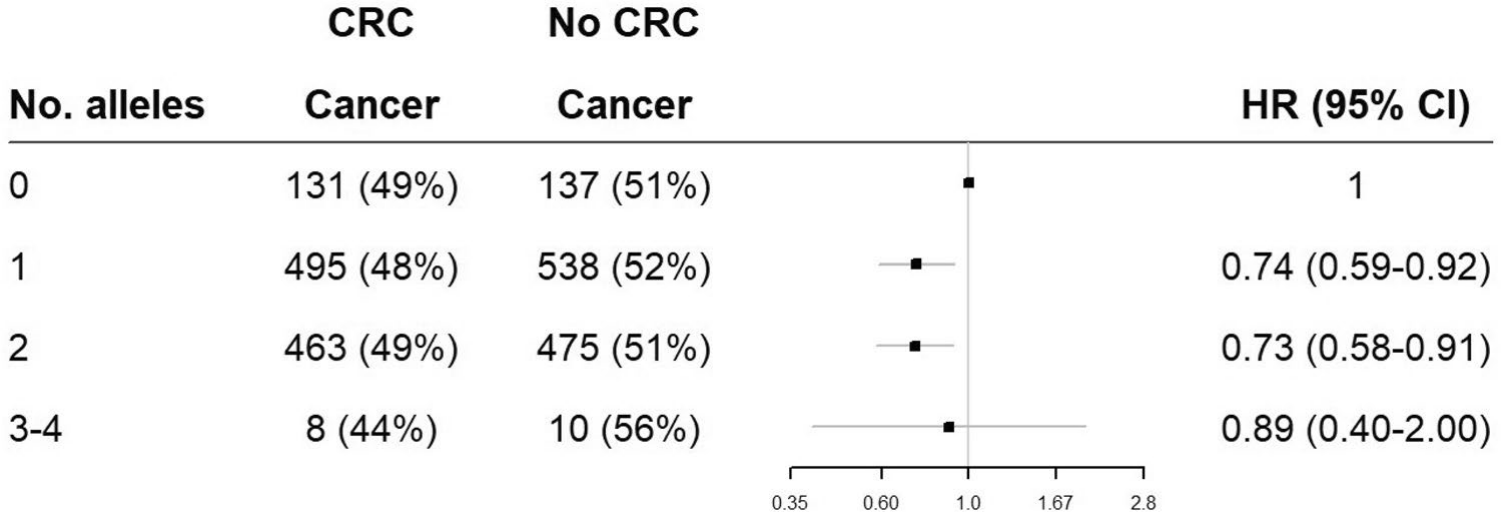
Displays the aggregated effect of protective alleles C (A1298C (rs1801131)) and T (C677T (rs1801133)) hazard ratios for risk of CRC in 0, 1, 2 and 3–4 protective alleles.

The aggregated effect of combined protective alleles from the two SNPs was explored. Due to low numbers of LS individuals carrying 3 or 4 protective alleles, these were combined into one group (3–4 alleles). A later age of onset of CRC was seen for the LS individuals with 3–4 protective alleles, but this was not significantly different due to the adjusted significance threshold (*p* = 0.04). Cox regression analysis showed that LS individuals with some protective alleles were significantly less likely to develop CRC than those with no protective allele. Having one protective allele was associated with a 26% reduction in risk (HR 0.74 (CI 0.59–0.92), *p* = 0.006), and having two protective alleles, a 27% reduction (HR 0.73 (CI 0.58–0.91), *p* = 0.006). However, having 3–4 protective alleles conferred no benefit (HR 0.89 (CI 0.40–2.00), *p* = 0.8), see Table 7 and Fig. 3.

**Table 7:** Displays results from Cox Regression analysis using the combined number of protective alleles for A1298C (rs1801131) and C677T (rs1801133) from the CRC sample cohort adjusted for gene, gender, and country.

Crude			Adjusted	
Gene	HR (95%CI)	*p*-value	HR (95%CI)	*p*-value
0	1		1	
1	0.73 (0.58–0.91)	0.006	0.74 (0.59–0.92)	0.006
2	0.78 (0.62–0.98)	0.032	0.73 (0.58–0.91)	0.006
3–4	0.55 (0.25–1.24)	0.150	0.89 (0.40–2.00)	0.800

## Discussion

Few studies have investigated the modifying effect of *MTHFR* SNPs on the risk of CRC in LS individuals, and their results are conflicting^19–21^. In this analysis, we aimed to verify previous findings to determine the modifying effect of *MTHFR* polymorphisms on LS expression by increasing the size of the analyzed cohort. The current study explores the role of two common *MTHFR* SNPs, A1298C (rs1801131) and C677T (rs1801133), and their effect on cancer risk in individuals with a molecular genetic diagnosis of LS.

These SNPs are alleged to be involved in the development of cancer, especially CRC, by altering *MTHFR* activity, which in turn reduces the silencing of tumour suppressor genes and increases the availability of nucleotides for DNA synthesis and repair, thereby protecting against early-onset cancer in LS^15,21^. The current study shows that these SNPs affect CRC risk but not LS cancer risk as a whole. The effect of C677T (rs1081133) is well established, with the variant allele resulting in a thermolabile enzyme with 65% (CT) and 30% (TT) enzyme activity, respectively, compared to wildtype genotype (CC)^23,25^. Several studies have found that LS individuals carrying one or more variant alleles of this SNP have a reduced risk of CRC^19–21, 26–32^. For A1298C (rs1081131), the reduction in MTHFR activity results in an enzyme with 85% activity for the AC genotype and 70% for the CC genotype compared to the AA genotype ^33,34^. Research on A1298C (rs1801131) and cancer risk display inconclusive association results and studies are often limited by small sample size^26,27, 32, 35^. However, some studies suggest that harbouring one or two C alleles on A1298C protects against developing CRC^21,29, 32^.

The current study shows LS cohorts consistent with published literature; individuals carrying germline *MSH6* pathogenic variants have a reduced risk of developing cancer compared to carriers of *MLH1* and *MSH2* pathogenic variants^4,5, 7, 8, 11, 12^.

Our findings display that irrespective of the mutated MMR gene, individuals with the AC genotype of the A1298C (rs1801131) SNP have a significantly reduced risk of developing CRC (17%) compared to those individuals with the AA genotype. The heterozygote AC genotype has previously been shown to reduce the risk of CRC^21,29, 32^, supporting the protective effect of the C allele. Individuals with the CC genotype also have a 22% reduced risk of CRC compared to the AA genotype, but this reduction was not statistically significant. Our results are similar to those of other studies^24,32, 35, 36^. Still, controversial results have been published showing an increased risk for genotype CC^19,27^, which was not confirmed in the current analysis. The small sample size in this group in the current study, reflected in the wide confidence interval, likely affected our power to estimate this effect.

Furthermore, we found that individuals with germline pathogenic variants in *MLH1* and the CC genotype of A1298C (rs1801131) had a significantly reduced risk of developing CRC (39%) compared to the rest of the cohort, indicating that the underlying germline MMR variant is important when looking at the modifying effects of *MTHFR* polymorphisms. These genotypes will be of even more interest once polygenetic risk scores become better defined. Our findings also showed that individuals with *MSH2* pathogenic variants and A1298C (rs1801131) genotype AC had a significantly reduced risk of developing CRC (26%) compared to individuals with *MSH2* pathogenic variant genotype AA, demonstrating that the heterozygote genotype has the best protective effect for these individuals. MTHFR is an important folate-metabolising enzyme that regulates DNA methylation and synthesis. Increased MTHFR activity has been theorised to result in earlier CRC onset, owing to the hypermethylation of tumour suppressor genes and the depletion of nucleotides available for DNA synthesis and repair. A limitation of our study was the inability to account for lifestyle and environmental factors, particularly folate status. It has been well established that adequate dietary folate consumption reduces cancer risk due to the hypermethylation of oncogenes^37^.

In conclusion, our study explored the association between *MTHFR* polymorphisms C677T (rs1801133) and A1298C (rs1801131) and the risk of developing CRC in LS individuals. We have shown that two genotypes (AC and CC) of SNP A1298C might have a protective effect on CRC development that differentiates between *MLH1* and *MSH2* germline variant carriers, which can explain some of the previous inconsistencies in results for this SNP and risk of CRC in LS individuals. In addition, we show that an aggregated effect of protective alleles from the two SNPs combined reduces the risk of CRC. Our study suggests that *MTHFR* genotypes, together with the underlying germline MMR gene, might be useful in an algorithm predicting the risk of developing CRC for individuals diagnosed with LS. The current study may also provide guidance for CRC risk estimation in LS individuals and contribute to reducing the current health, social and economic burden of cancer development in LS individuals.

## Data Availability

The data that support the findings of this study are not openly available due to reasons of sensitivity, privacy and custodianship but will be made available from the corresponding author upon reasonable request.
